# The memory remains

**DOI:** 10.18632/aging.101408

**Published:** 2018-03-29

**Authors:** Matthias Altmeyer

**Affiliations:** 1 Department of Molecular Mechanisms of Disease, University of Zurich, CH-8057, Zurich, Switzerland

**Keywords:** cell cycle, replication stress, DNA damage, p53, p21

Too much stress is unhealthy, it causes us to age faster and contributes to the development of diseases. However, certain tasks in life need to be completed within a given time-frame, and this necessitates good organizational skills and an efficient mode of operation. Not always can stress be avoided in such situations, and arguably more important than stress avoidance is proper time management and a good balance between labor-intense periods and more relaxed phases, which allow time for recovery.

What could well be a guideline for a healthy work-life balance in the 21^st^ century also accurately describes the challenges faced by actively dividing cells, the smallest units of life and the workhorses of our body. During cell cycle progression, these have to coordinate multiple important tasks in a limited amount of time and with limited resources. While such multi-tasking is vital for proper cell functioning and required to ensure timely completion of the cell division cycle, it also comes with an increased stress risk. DNA replication stress, for instance, is related to the intricacy associated with the duplication of a vast amount of genetic information in a relatively short time window, with complicating processes such as transcription happening simultaneously. No wonder that S-phase progression is often stressful for cells and allows little opportunities to breathe and relax. How then do cells maintain a healthy balance?

One way could really be to alternate stressful phases with more relaxed ones, pushing through difficult periods to meet time-sensitive deadlines, and have them be followed by periods of recovery and repair. Intriguingly, accumulating evidence from multiple research groups suggests that this could indeed be the modus operandi of rapidly dividing cells. As a consequence, different cell cycle phases actually feed into each other rather than representing strictly separated entities [1,2]. For example, stress experienced during S-phase frequently manifests as DNA lesions in the next G1 phase of the cell cycle, suggesting that cells compromise on genome maintenance during S-phase progression, refrain from being perfectionists, and instead use subsequent cell cycle phases to restore genome integrity. What may seem like a dangerous game could in fact reduce the stress to tolerable levels and thereby enhance cellular fitness.

In parallel work it was found that cells exiting from mitosis can either enter a state, which primes them for rapid cell cycle re-entry and S-phase commitment, or an alternative state of prolonged G1 duration [3]. Four research articles published in 2017 now provide a rationale for this variability in S-phase commitment and link it back to the extent of stress experienced in the previous cell cycle [4–7]. All four studies come to the conclusion that cells do not erase their history when new cells are being born during mitosis, but that instead newly born cells inherit stress signals from the previous cell generation, which in turn determines their resting time in G1 before they start a new round of replication. In other words, cells seem to possess a signaling memory, which presumably allows them to cope better with cellular stress. The four studies also converge on a role of the cell cycle regulators p53 and p21 in controlling G1 duration, in line with their function as natural barrier against cancer development. Consequently, cells lacking p53 fail to prolong their G1 phase and, when having inherited DNA lesions from the previous cell cycle, enter S-phase with an elevated damage load [6]. While it is still poorly understood how DNA lesions inherited from the parental cell generation are eventually resolved, these results emphasize that resting time, even for the ‘young’ generation, is important for stress resilience, and that continuously high levels of stress without the opportunity to pause and recover may undermine genome stability. Oncogene activation, in conjunction with loss of p53 function, may lead to such an unhealthy condition, in which over multiple cell generations stress inheritance eventually causes stable genetic alterations in the offspring and thereby drives cancer development.

## References

[1] Lukas J, et al. Nat Cell Biol. 2011; 13:1161–69. 10.1038/ncb2344 21968989

[2] Mankouri HW, et al. EMBO J. 2013; 32:2661–71. 10.1038/emboj.2013.211 24065128 PMC3801442

[3] Spencer SL, et al. Cell. 2013; 155:369–83. 10.1016/j.cell.2013.08.062 24075009 PMC4001917

[4] Arora M, et al. Cell Reports. 2017; 19:1351–64. 10.1016/j.celrep.2017.04.055 28514656 PMC5533606

[5] Barr AR, et al. Nat Commun. 2017; 8:14728. 10.1038/ncomms14728 28317845 PMC5364389

[6] Lezaja A, Altmeyer M. Cell Cycle. 2018; 17:24–32. 10.1080/15384101.2017.1383578 28980862 PMC5815429

[7] Yang HW, et al. Nature. 2017; 549:404–08. 10.1038/nature23880 28869970 PMC6544019

